# Cytotoxicity of Peruvian propolis and *Psidium guajava* on human gingival fibroblasts, PBMCs and HeLa cells

**DOI:** 10.12688/f1000research.110352.1

**Published:** 2022-04-19

**Authors:** Pablo Alejandro Millones-Gómez, Myriam Angélica De la Garza-Ramos, Victor Hugo Urrutia-Baca, Humberto Carlos Hernandez-Martinez, David Alejandro Hernández Marín, Carlos Alberto Minchón Medina

**Affiliations:** 1 Escuela de Medicina Humana, Universidad Señor de Sipán, Chiclayo, 14000, Peru; 2 Facultad de Odontologia, CIDICS/UOIE, Universidad Autónoma de Nuevo León, Monterrey, 64000, Mexico; 3 Facultad de Ciencias Biológicas, Laboratorio de Inmunología y Virología/ CIDICS/UOIE, Universidad Autónoma de Nuevo León, Monterrey, 64000, Mexico; 4 Departamento de Microbiología, Centro de Ciencias Básicas, Universidad Autónoma de Aguascalientes, Aguascalientes, 20100, Mexico; 5 Department of Statistics, Faculty of Physical Sciences and Mathematics, Universidad Nacional de Trujillo, Trujillo, 13001, Peru

**Keywords:** Cytotoxicity, Viability, Propolis, Guava

## Abstract

It is indisputable that every day it is demonstrated that natural products present diverse therapeutic benefits, which has boosted their incorporation within various products for clinical use. However, this must be accompanied by knowledge of their effect on cell lines to ensure their use is safe. The objective of this study was to evaluate the cytotoxic effect of two ethanolic extracts based on Peruvian natural products, on three human cell lines. Cervical cancer cell lines (HeLa), human gingival fibroblasts (HGF-1 - ATCC CRL-2014) (HGF-1) and peripheral blood mononuclear cells (PBMCs) were cultured and subsequently treated with preparations of ethanolic extracts of propolis (EEP) and Psidium guajava (EEG) from a concentration of 50 mg/mL to 0.024 mg/mL, by the 3-(4,5- dimethylthiazol-2-yl)-2,5-diphenyltetrazole bromide reduction assay. At a concentration of 0.24 mg/mL EEG, viability of 99.7±1.24%, 99.8±2.2% and 99.7±2.7% was observed in HeLa, HGF-1 and PBMCs, respectively; >90% cell viability values were observed with EPP at 0.024 mg/mL, with HGF-1 showing the highest viability (96.9±1.15%). A dose-dependent effect was observed for both extracts with a decrease in cell viability as concentrations increased (up to 50 mg/mL). EEP and EEG extracts at low concentrations do not show cytotoxicity in human cell lines, these findings are an advance in the preclinical evaluation on their safety and open a continuity to further studies for their potential applications in dentistry and medicine.

## Introduction

Since time immemorial, man has tried to mitigate his ailments and prolong his life. This fact has been observed since there have been historical records, from civilization to civilization, until today. ^1^ Even so, man in the 21st century has not been able to avoid death by limiting himself to mitigating symptoms of diseases and avoiding the development of others. ^1^^,^^2^

In times when man only had at his disposal the resources that the planet gave him, he sought in these the tools to reduce physical pain and avoid death. Among the resources most exploited by different cultures throughout history are mineral, animal and vegetable resources. Until the middle of the 20th century, these were the therapeutic resources par excellence. ^2^^,^^3^

Among the kingdoms of nature that contribute, to this day, to reducing symptoms and preventing diseases, the plant kingdom stands out. ^4^ Plants, thanks to their marvelous and complex metabolism, constitute a true chemical arsenal. Of which only a third is currently known, considering the variety of existing species worldwide, without considering those species already extinct. ^3^^,^^4^

Each region of the world developed its own way of healing from medicinal plants, which is unique and characteristic since species endemic to regions were used. ^4^ Over time, these local characteristic therapies came to form the so-called traditional medicine and, when being preserved by the native peoples, is sometimes called aboriginal or autochthonous medicine, as well as traditional or autochthonous recipes ^4^ that group together uses, forms of preparation, administration, dosage, among other modern pharmacological parameters. This is because our therapeutic reality today is governed by synthetic chemistry, but what few people know is that these successful molecules that cure are nothing more than improved copies of chemical substances that nature spontaneously created. ^4^

One of the most studied products is propolis, ^5^ which is composed of approximately 50% to 55% resins and balsams, 30% to 40% wax, 10% to 15% essential oils, 5% pollen and 5% minerals. ^5^ In its components we can mention that it has phenolic compounds: Flavonoids, flavones, isoflavones and flavonones in 50%, ^5^ which inhibit bacteria and fungi. ^6^ The amount of flavonoids confers the antibacterial power to propolis. This quantity depends on the flora surrounding the bee hives. ^5^ Its antibacterial action mechanism is given by the inhibition of cell division, DNA disruption, disorganization of the cytoplasmic membrane and inhibition of cell wall synthesis, causing partial bacteriolysis and inhibiting protein synthesis. ^5^^,^^6^

Guava ( *Psidium guajava*) is a fruit native to Central America and the Caribbean, belonging to the Myrtaceae family, distributed in the tropics and subtropics around the world. Guava fruits stand out among tropical fruits not only because of their good organoleptic characteristics (flavor and aroma) but also nutritionally, they are a source of vitamin A, B1, B3 and C, fiber, minerals such as potassium, calcium, iron and phosphorus. ^7^ The guava also has a relevant content of lycopene, an important carotenoid with therapeutic properties, so it has been widely studied. ^7^^,^^8^

Cytotoxic evaluation, as the main factor of biocompatibility, is determined by the cell cultures to be selected for *in vitro* toxicity testing. ^9^ Continuous and real-time monitoring allows label-free assessment of cell proliferation, viability and cytotoxicity, revealing the physiological status of the cells. ^8^ To evaluate the efficacy of natural products, it is not enough to measure their therapeutic effect, but one must be sure that they do not cause deterioration of constituent cells. The aim of this study was to evaluate the cytotoxic effect of ethanolic extracts of propolis and *P. guajava* on HELA cell lines, human gingival fibroblasts (HGF-1) and peripheral blood mononuclear (PBMCs) cells.

## Methods

### Preparation of ethanolic extracts

Propolis and *P. guajava* samples were collected by researchers in the Oxapampa valley, Pasco, Peru following the methodology described by Millones *et al.* ^5^^,^^8^ and subsequently refrigerated until processed. After they were removed from refrigeration they were left for two hours to allow for them to reach room temperature. Once they had reached room temperature they were macerated Once room temperature was reached, they were macerated with a volume of 100 ml of absolute ethanol for every 10 grams of propolis sample, it was then left at room temperature for 24 hours. Then, the macerate was filtered using a 20 cm diameter glass funnel with sterile cotton; the filtered sample was collected in a glass refractory to finally be taken to an extraction hood so that the ethanol present in the extract evaporates completely and only a pasty mass remains. This step was performed two more times until the samples were observed to be discolored. Finally, they were stored in glass containers covered with aluminum foil to avoid degradation. ^10^

### HeLa cell line preparation

The HeLa cell line (ATCC, Manassas, VA, USA) (RRID:CVCL_0058) was cultured in a Petri dish with a 35 mm diameter glass bottom (MatTek Corporation, Ashland, MA, USA, CAT#: P DCF OS 30). The cell line was cultured in Eagle’s Minimum Essential Medium **(**Gibco) with 4% PBS (phosphate buffered saline) at 37°C in 5% CO _2_ and 95% air. ^5^ The cells were incubated for 30 min in 1 mL of dye solution in Hank’s Balanced Salt Solution (HBSS, Thermo Fisher Scientific)) (100 nM Mitotracker Red FM) at 37°C, 5% CO _2_. After incubation, the cells were washed three times with HBSS buffer. ^11^

### Human gingival fibroblasts culture

Human gingival fibroblasts (HGF-1 - ATCC CRL-2014) (HGF-1) were obtained from the American Type Culture Collection (ATCC) and cultured in Dulbecco’s modified Dulbecco’s medium containing glucose (DMEM; Sigma-Aldrich, St. Louis, MO, USA) supplemented with 4 mM L-glutamine (Sigma-Aldrich), 1% penicillin, streptomycin (Sigma-Aldrich) and 10% (vol/vol) heat-inactivated fetal bovine serum (FBS; Sigma-Aldrich). Cells were incubated at 37°C in a 5% CO _2_ atmosphere in an incubator (Cytomat 2C450S; Thermo Fisher Scientific), and were fed every 48 hours and subcultured every 5 days at a 1:3 ratio using trypsin-EDTA (0.05%; Sigma-Aldrich) for 3 minutes at 37°C. ^12^

### PBMCs culture

PBMC cells were isolated by Ficoll density gradient centrifugation (TBD, Shanghai, China) and cultured in RPMI-1640 medium with 10% fetal bovine serum and placed in a humidified incubator (Thermo CO _2_ incubator, 311, USA) at 37°C, 5% CO _2_ and 95% humidity. The medium was changed once every 24 hours. ^13^^–^^16^

### Cytotoxicity assays

To evaluate the cytotoxic effect, cell viability methodology was performed in microplate with 3-(4,5-dimethylthiazol-2-yl)-2,5-diphenyltetrazole bromide (MTT). ^10^ For this, confluent cell cultures of (80-100%) contained in 25 cm ^2^ flasks were started, the medium was discarded using a 5mL serological pipette, two washes were performed to the cell layer with PBS solution (0.004% Ethylene diamine tetracetic acid), 1 mL of Trypsin-EDTA (0.05%) was immediately added and incubated at 37°C for 15 min. A 20 μL sample was taken and 20 μL of 0.4% trypan blue (Gibco, Carlsbad, CA, USA) was added to perform a cell count in a Neubauer chamber (Sigma-Aldrich, model: Bright-Line ^TM^ Hemacytometer, catalog number: Z359629) to adjust the cell concentration to 5×104 cells per reaction with Dulbecco’s Modified Eagle’s medium (d-MEM) supplemented with 10% fetal bovine serum. Then 50 μL of the cell suspension was seeded, incubated at 37° C, 5% CO _2_ in an incubator (Cytomat 2C450S; Thermo Fisher Scientific) until confluence was obtained (after 48 hours), then different concentrations of the natural products ^5^ (50 to 0.024 mg/mL) were applied and the plates were incubated for 48 hours. Only d-MEM culture medium was used as a negative control and as a positive control.

The medium used was then discarded and the cell layer was washed with PBS solution (0.004% EDTA) and 50 μL of d-MEM culture medium was added.

### MTT assay

To each well, 20 μL of MTT (5 mg/mL) was added to each culture and then the cultures were incubated for four hours. After this time, the medium was aspirated. 200 μL of dimethyl sulfoxide was added to dissolve the formazan crystals; the plate was left in agitation for 15 minutes microplate shaker (Lab-Line Instrument Inc. Melrose Park, IL) and shaken at 120 revolutions per minute to ensure complete dissolution. Finally, the plate was read at 570 nm on a Smart Spectophotometer plus reader (1705061, Bio-Rad, Hercules, CA, USA).

### Statistical analysis

The experimental data were analyzed using nonlinear regression with the Gompertz model to evaluate the effect according to the concentration of the doses used, whose equation is given by: ^32^

$$y=\alpha \;\exp\left\{-\beta e^{-k\;x}\right\}$$

Where, y is the cell viability, x the administered concentration of each product (mg/mL), and α, β, and k are the parameters of the model. Comparison of the cytotoxic effect of propolis and *P. guajava* was performed using analysis of covariance, which in addition to the product includes the concentration administered. Graphical presentation was prioritized to highlight some analyses. The analyses were performed with Excel (Microsoft Corporation, US, 2019) (RRID:SCR_016137) and SPSS version 26 (IBM, US, 2019) (RRID:SCR_016479).

## Results

**Figure 1.**
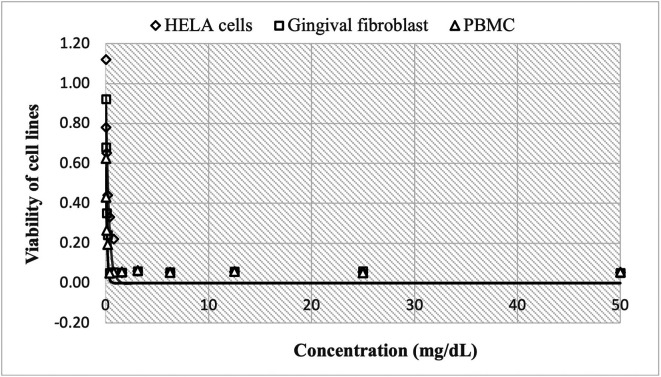
Viability of cell lines by effect of concentrations of ethanolic extracts of propolis. The cytotoxic effect of Peruvian propolis is shown in Figure 1, with high cell viability at concentrations of 0.24 mg/mL, reaching 1.120±0.012 *Henrietta Lacks* cells (HELA cells), 0.922±0.011 human gingival fibroblasts (HGF-1) and 0.624±0.002 *peripheral blood mononuclear cells* (PBMCs cells), decreasing rapidly to 0.052±0.002, 0.051±0.001 and 0.055±0.001, respectively as concentrations increase up to 50 mg/mL. The observed trend shows that cell growth decreases in a non-linear fashion as the dose of Peruvian propolis administered increases.

The cytotoxic effect of Peruvian propolis is shown in Figure 1, with high cell viability at concentrations of 0.24 mg/mL, reaching 1.120±0.012 HBA cells, 0.922±0.011 HGF-1 and 0.624±0.002 PBMCs cells, decreasing rapidly to 0.052±0.002, 0.051±0.001 and 0.055±0.001, respectively as concentrations increase up to 50 mg/mL. The estimated nonlinear Gompertz regression models were:

$$\text{HELA cells}:y=437294.952\;\exp\left\{-12.972\;e^{0.249\;x}\right\},R^{2}=0.927$$

$$\text{Gingival fibroblast}:y=186292.087\;\exp\left\{-12.027\;e^{0.782\;x}\right\},R^{2}=0.960$$

$$\text{PBMC}:y=206045.920\;\exp\left\{-12.601\;e^{0.618\;x}\right\},R^{2}=0.917$$

**Figure 2.**
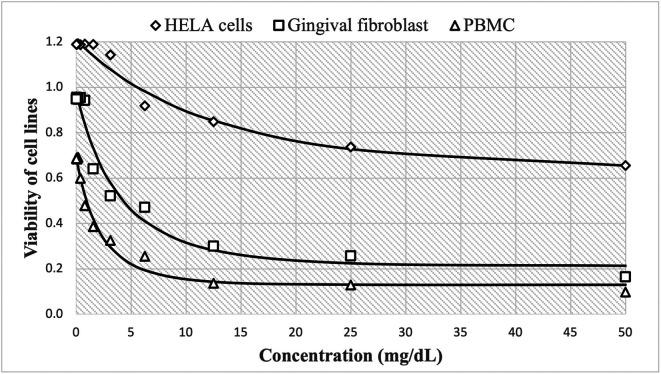
Viability of cell lines by the effect of concentrations of ethanolic extracts of *P. guajava.* The cytotoxic effect of guajava is shown in Figure 2, with high cell viability at concentrations of 0.24 mg/mL, reaching 1.190±0.015 *Henrietta Lacks* cells (HELA cells), 0.948±0.020 human gingival fibroblasts (HGF-1) and 0.685±0.019 *peripheral blood mononuclear cells* (PBMCs cells). These decreased more slowly to 0.656±0.019, 0.165±0.020 and 0.099±0.002, respectively as concentrations increase up to 50 mg/mL. Similarly, the observed trend shows that cell growth decreases in a non-linear fashion as the dose of guajava administered increases.

On the other hand, the cytotoxic effect of Peruvian *P. guajava* is shown in Figure 2, with high cell viability at concentrations of 0.24 mg/mL, reaching 1.190±0.015 HBA cells, 0.948±0.020 HGF-1 and 0.685±0.019 PBMCs cells. These decreased more slowly to 0.656±0.019, 0.165±0.020 and 0.099±0.002, respectively as concentrations increase up to 50 mg/mL. The estimated nonlinear Gompertz regression models were:

$$\text{HELA cells}:y=437294.952\;\exp\left\{-12.972\;e^{0.249\;x}\right\},R^{2}=0.974$$

$$\text{Gingival fibroblast}:y=186292.087\;\exp\left\{-12.027\;e^{0.782\;x}\right\},R^{2}=0.970$$

$$\text{PBMC}:y=206045.920\;\exp\left\{-12.601\;e^{0.618\;x}\right\},R^{2}=0.982$$

The goodness of fit of the Gompertz curves to the cytotoxic effect on the cell lines of both products is shown by the coefficient of determination (R ^2^), this value is above 90% for all curves.

The cytotoxic effect of propolis and *P. guajava* on the cell lines was compared using analysis of covariance, which are shown in Table 1. In each of the cell lines, differences in the cytotoxic effect between both products were observed (p<0.01 in each of the lines); likewise, the linear effect of the concentrations used for each of the products was observed (p<0.01 in each of the lines), even though the non-linear effect was verified by means of the Gompertz model.

**Table 1.** Analysis of covariance cytotoxic effect of ethanolic extract concentrations of propolis and *P. guajava* on cell line viability.

Cell line	Source of variation	Sum of squares	Degrees of freedom	Mean square	F	p
HELA cells	Model	3.847	2	1.923	33.170	0.000
	Products	3.200	1	3.200	55.185	0.000
	Concentrations	0.647	1	0.647	11.155	0.003
	Error	1.218	21	0.058		
	Total	5.064	23			
Gingival fibroblast	Model	1,899	2	0.949	14.640	0.000
	Products	1.225	1	1.225	18.891	0.000
	Concentrations	0.674	1	0.674	10.389	0.004
	Error	1.362	21	0.065		
	Total	3.260	23			
PBMC	Model	0.745	2	0.373	11.200	0.000
	Products	0.428	1	0.428	12.867	0.002
	Concentrations	0.317	1	0.317	9.533	0.006
	Error	0.699	21	0.033		
	Total	1.444	23			

The cytotoxic effect of propolis and *P. guajava* on the cell lines was compared by analysis of covariance, which are shown in Table 1. In each of the cell lines, differences in the cytotoxic effect between propolis and *P. guajava* were observed for *Henrietta Lacks* cells (HELA cells) (F=55.185, p=0.000 <0.05), gingival fibroblast (F=55.185, p=0.000 <0.05), and *peripheral blood mononuclear cell* (PBMC) (F=12.867, p=0.002 <0.05). Likewise, the linear cytotoxic effect of product concentrations on the viability of HELA (F=11.155, p=0.003 <0.05), gingival fibroblast (F=10.389, p=0.004 <0.05), and PBMC (F=9.533, p=0.006 <0.05) cells was observed, although the non-linear effect was verified by the Gompertz model.

**Figure 3.**
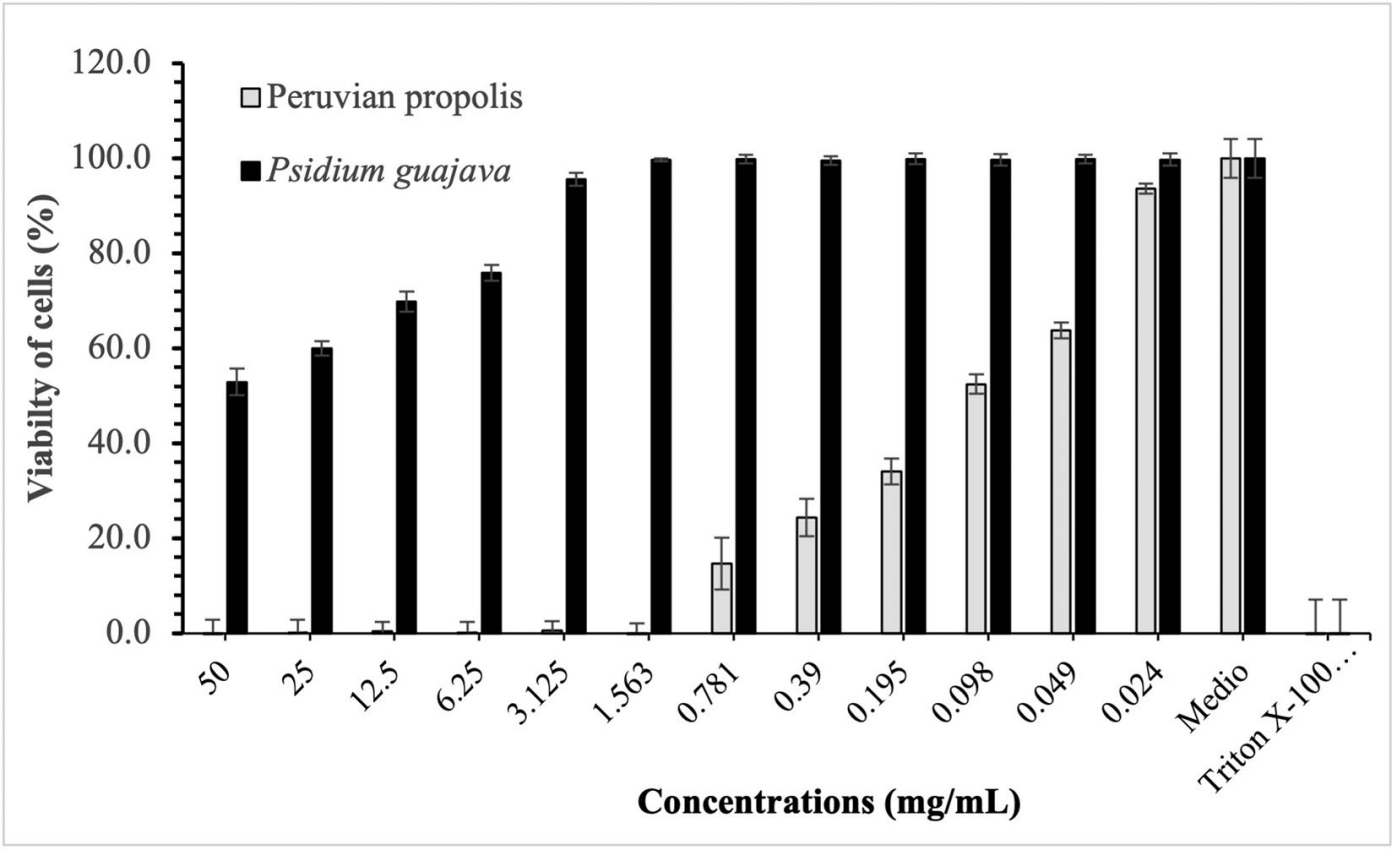
Viability of HELA cells by effect of concentrations of ethanolic extracts of propolis and *P. guajava.* Figure 3 compares the cytotoxic effect of propolis and *P. guajava* concentrations and Triton X-100 on *Henrietta Lacks* cells (HELA cells) viability (%), showing the average and the corresponding standard deviations. With propolis, the growth of HELA cells remains in control with doses of 50-1,563 mg/mL, increasing very rapidly with smaller doses. In contrast, with guajava, cell growth is already more than 50% at doses of 50 mg/mL, growing rapidly, and reaching maximum levels at doses of 1,563 mg/mL or lower.

**Figure 4.**
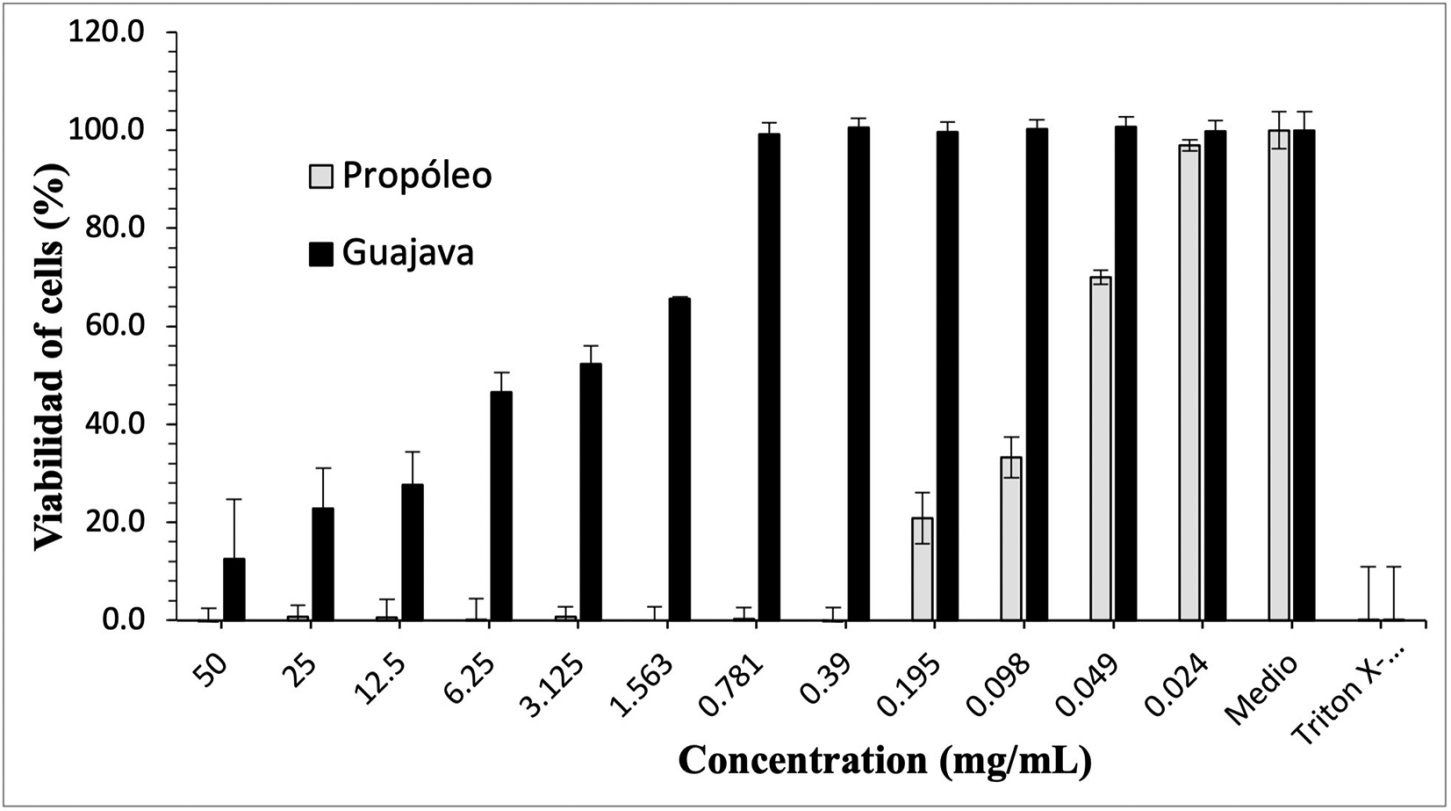
Viability of human gingival fibroblasts by effect of ethanolic extracts concentrations of propolis and *P. guajava.* The growth of human gingival fibroblasts remains in control with propolis doses of 50-0.39 mg/mL, with rapid growth at lower doses; in contrast, with gujava, while at doses of 50 mg/mL cell growth was pc more than 10%, it begins to increase considerably up to doses of 0.781 mg/mL, at which it reaches maximum growths.

**Figure 5.**
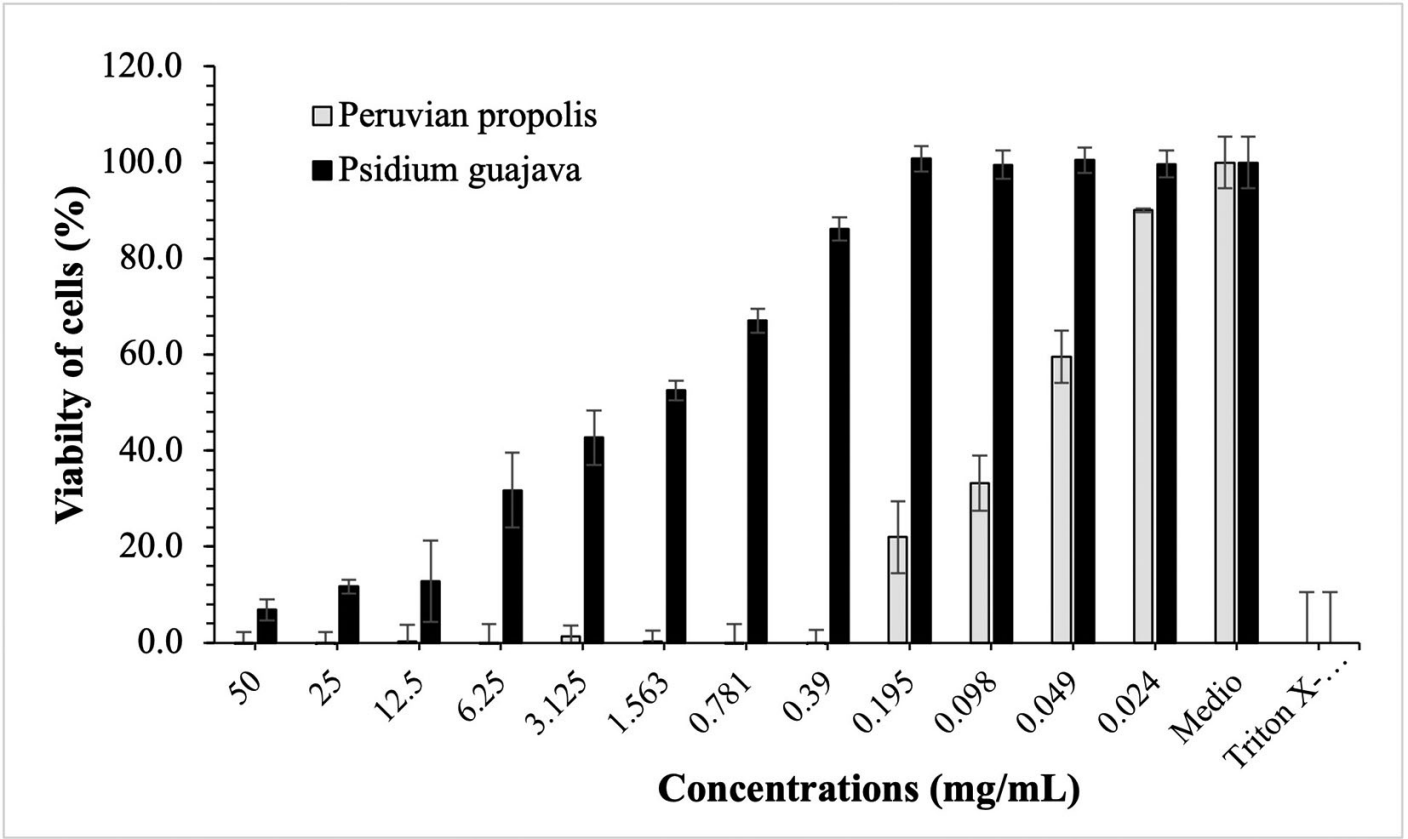
Viability of peripheral blood mononuclear cells (PBMCs) by effect of concentrations of ethanolic extracts of propolis and *P. guajava.* The growth of *peripheral blood mononuclear cells* (PBMCs cells) remains in control with propolis doses of 50-0.39 mg/mL, with rapid growth at lower doses; on the contrary, with guajava, although at a dose of 50 mg/mL cell growth was small, it begins to increase considerably up to doses of 0.195 mg/mL, at which it reaches maximum growth.

Figures 3- 5 show the cytotoxic effect of propolis and *P. guajava* on the cell lines as a percentage of cell viability, established from the medium controls and Triton X-100, being notorious the differences in the effect between both products, as already shown.

## Discussion

The aim of this study was to evaluate the cytotoxic effect of ethanolic extracts of propolis and *P. guajava* on HELA cell lines, HGF-1 and PBMCs cells. The results showed high cell viability at concentrations of 0.24 mg/mL, decreasing rapidly as concentrations increased up to 50 mg/mL.

The cytotoxicity assay revealed that Peruvian propolis and guajava extracts at lower concentrations can work safely on the fibroblast cell line. However, it is important to recognize that since this is an *in vitro* assay, this value may vary if other types of cell lines are used. The results obtained in the cytotoxicity assay, add to the increasingly abundant information reported by other research groups that have wanted to address this issue but focusing on alcoholic extracts, ^17^^–^^19^ and are therefore important. The content of phenols and flavonoids obtained in the aqueous extract of propolis is lower than that reported by other research groups ^20^ however, it should be noted that the extraction methods were different and as mentioned, these are determinant for the preparation of the extract. To determine which methodology is more efficient, the same propolis sample should be used, ^21^^–^^24^ due to the high variability in the composition that this product may have with respect to its region of origin. ^24^

As for *P. guajava*, it showed similar toxicity in the three cell lines. Some studies report a higher amount of active metabolites in the peel with respect to guava pulp, and also report a better antioxidant capacity *in vitro.* ^24^^,^^25^ With the cytotoxic effect shown in HeLa cells, the world list of plants with potential for cases of cervical neoplasia published is increased, the results obtained in the study contribute to corroborate the properties traditionally attributed to these plants and highlight species of the Peruvian medicinal flora as a source of substances for the treatment of cancer. ^25^

Despite the high cytotoxicity shown by most of the propolis samples against the cell lines studied, the samples also showed toxicity to HGF-1 culture. Ling *et al*. ^26^ have also investigated the cytotoxicity of Brazilian red propolis extracts for two tumor cell lines (Hep-2 and HeLa) and for normal human embryonic epithelial kidney (Hek-293), also reporting a higher IC _50_ value for Hek-293 compared to the tumor cell lines.

Although both propolis and guava seem to exert a potential on PBMCs cells, only a few studies have been performed in this field of research. ^27^^–^^30^ Despite this, our preliminary data suggest that both products could present a modulatory action on the immune response opening perspectives for further research in this field.

## Conclusions

EEP and EEG at low concentrations do not show cytotoxicity in human cell lines and their effect is dose dependent. Our findings are an advance in the preclinical evaluation of natural extracts from Peru on their safety and open a continuity to further studies for their potential applications in dentistry and medicine. Despite our positive data, further study is required to evaluate the usefulness of these extracts.

## Data availability

### Underlying data

Mendeley: Cytotoxicity database. https://doi.org/10.17632/yt4h7h9cvv.1 ^31^

This project contains the following underlying data:

- citotoxicidad.sav (raw data)

### Extended data

Mendeley: Cytotoxicity database. https://doi.org/10.17632/yt4h7h9cvv.1 ^31^

This project contains the following extended data:

- database.xlsx (Processed data)

Data are available under the terms of the Creative Commons Attribution 4.0 International license (CC-BY 4.0).
