# Supplementary material for: Cytotoxicity of Peruvian propolis and Psidium guajava on human gingival fibroblasts, PBMCs and HeLa cells
Source: F1000Res. 2022 Aug 16;11:430. Originally published 2022 Apr 19. [Version 2] doi: 10.12688/f1000research.110352.2 (PMC9386296; doi:10.12688/f1000research.110352.2)
Supplement: Peer review history

# Peer review: Reviewer response for version 2

**DOI:** 10.5256/f1000research.137410.r147645

**Published:** 2022-08-17

**Authors:** Orlando Torres Garcia

**Affiliations:** 1 Faculty of Veterinary Medicine and Zootechnics, Universidad Antonio Nariño, Bogotá, Colombia; 2 Faculty of Sciences, Javeriana University, Bogotá, Colombia

The document is interesting and presents two compounds of natural origin (Propolis and Psidium guajava) with potential for medical and dental application.

Is the work clearly and accurately presented and does it cite the current literature?

Yes

If applicable, is the statistical analysis and its interpretation appropriate?

Yes

Are all the source data underlying the results available to ensure full reproducibility?

Yes

Is the study design appropriate and is the work technically sound?

Partly

Are the conclusions drawn adequately supported by the results?

Yes

Are sufficient details of methods and analysis provided to allow replication by others?

Yes

Reviewer Expertise:

Immunology and Immunogenetics, microbiology.

I confirm that I have read this submission and believe that I have an appropriate level of expertise to confirm that it is of an acceptable scientific standard.

# Peer review: Reviewer response for version 2

**DOI:** 10.5256/f1000research.137410.r147646

**Published:** 2022-08-17

**Authors:** Tania Valentina Rosales Cifuentes

**Affiliations:** 1 Universidad Nacional Mayor de San Marcos, Lima, Peru

Is the work clearly and accurately presented and does it cite the current literature?

Yes

If applicable, is the statistical analysis and its interpretation appropriate?

Yes

Are all the source data underlying the results available to ensure full reproducibility?

Yes

Is the study design appropriate and is the work technically sound?

Yes

Are the conclusions drawn adequately supported by the results?

Yes

Are sufficient details of methods and analysis provided to allow replication by others?

Yes

Reviewer Expertise:

Natural products and microbiology

I confirm that I have read this submission and believe that I have an appropriate level of expertise to confirm that it is of an acceptable scientific standard.

# Peer review: Reviewer response for version 1

**DOI:** 10.5256/f1000research.121947.r144832

**Published:** 2022-08-09

**Authors:** Orlando Torres Garcia

**Affiliations:** 1 Faculty of Veterinary Medicine and Zootechnics, Universidad Antonio Nariño, Bogotá, Colombia; 2 Faculty of Sciences, Javeriana University, Bogotá, Colombia

In this article, the authors evaluated the cytotoxic effect of ethanolic extracts of propolis and *P. guajava* on HELA cell lines, human gingival fibroblasts (HGF-1), and peripheral blood mononuclear cells (PBMC), finding cell viability more significant than 90% at concentrations equal to or less than 0.24 mg/mL and 0.024 mg/mL for EEG and EPP, respectively. Within this perspective, the authors infer potential applications in medicine and dentistry.

**Main comments:**

1. In the study methodology, the authors use the HELA cell lines, human gingival fibroblasts (HGF-1), and peripheral blood mononuclear cells (PBMC); however, the reason for their selection they not mentioned in the text. I suggest the authors include some selection criteria for these cell lines used in work.
2. To determine the cytotoxicity, the authors use the ethanolic extracts of propolis and *P. guajava* at the concentration range of 0.024 to 50 mg/mL. What were the selection criteria for this range of concentrations?
3. Although the document achieves the objective of determining the highest concentrations of the two ethanolic extracts at which cell viabilities more significant than 90% are observed, it would also be interesting to calculate the IC50 of each extract on each cell line.

**Minor comment:**

1. In the last paragraph of the discussion, the authors state that their preliminary data suggest that both products could have a modulating action on the immune response. I suggest rethinking this inference because within the document's text, and mainly, neither in the objective nor in the methodology, and even less in the results, are not elements that allow this insinuation in the present study.

Is the work clearly and accurately presented and does it cite the current literature?

Yes

If applicable, is the statistical analysis and its interpretation appropriate?

Yes

Are all the source data underlying the results available to ensure full reproducibility?

Yes

Is the study design appropriate and is the work technically sound?

Partly

Are the conclusions drawn adequately supported by the results?

Yes

Are sufficient details of methods and analysis provided to allow replication by others?

Yes

Reviewer Expertise:

Immunology and Immunogenetics, microbiology.

I confirm that I have read this submission and believe that I have an appropriate level of expertise to confirm that it is of an acceptable scientific standard, however I have significant reservations, as outlined above.

# Peer review: Reviewer response for version 1

**DOI:** 10.5256/f1000research.121947.r135716

**Published:** 2022-05-23

**Authors:** Tania Valentina Rosales Cifuentes

**Affiliations:** 1 Universidad Nacional Mayor de San Marcos, Lima, Peru

**Title:** Its title is adequate, it consigns the most interesting part of the study referred to the cytotoxicity of propolis, using scientific terms keeping the coherence and sufficiency of the article's content.

**Summary:** Although it is true that natural products are beneficial, there are few studies that mention the safety of the clinical use of the product. The article emphasizes the importance of knowledge of cell toxicity with a clear and precise explanation of the adequate concentration to avoid the cytotoxic effect.

**Content:** The methods section is well explained, the cytotoxic assay of this study establishes which was the adequate procedure that provided the percentage of safe and reliable cell viability, being coherent with the results in Figure 1. I consider that establishing the detailed procedure as it is done in this study is important so that it is reproducible in future studies of regenerative medicine or tissue engineering.

**Introduction:** I appreciate the effort of the background search and its respective analysis, the introduction is very good, it makes a brief summary of the background making reference to the relevant medical literature, in the last paragraph it mentions the safety of the use of propolis without deterioration of the cells, I suggest, so that the reader can make a comparison and as interesting reading, to cite references 27 and 30, which are similar studies.

**Results:** The results of this study are accurate, achieving the objective to avoid cytotoxicity in human cell lines, recognizing that the effect is dose-dependent. Based on the experience of the researchers evidenced in previous publications, it can be observed that a line of research continues in search of new active principles that can offer a promising pharmacological product based on natural products. Although it is true that this is a preliminary study as indicated by the authors, it is important to point out that future research could consider procedures based on flow cytometry to corroborate the results obtained in the present study.

**Discussion: **The background information showed the therapeutic benefits of natural products, with propolis standing out from the two products, for its antimicrobial, anti-inflammatory and antioxidant effects, among others; however, complementary studies are required to verify its safety in clinical use. The article specifies its strengths and limitations by mentioning which is the ideal concentration to avoid cytotoxicity and that the extracts of Peruvian propolis and guava at lower concentrations can work safely in the fibroblast cell line; however, the authors recognize that since this is an in vitro assay, this value may vary if other types of cell lines are used, making a discussion with other studies carried out; they also suggest that both products could present a modulating action on the immune response, opening perspectives for future research in this field.

Is the work clearly and accurately presented and does it cite the current literature?

Yes

If applicable, is the statistical analysis and its interpretation appropriate?

Yes

Are all the source data underlying the results available to ensure full reproducibility?

Yes

Is the study design appropriate and is the work technically sound?

Yes

Are the conclusions drawn adequately supported by the results?

Yes

Are sufficient details of methods and analysis provided to allow replication by others?

Yes

Reviewer Expertise:

Natural products and microbiology

I confirm that I have read this submission and believe that I have an appropriate level of expertise to confirm that it is of an acceptable scientific standard.
